# Changes in cardiorespiratory status after transcatheter patent ductus arteriosus closure

**DOI:** 10.1038/s41372-025-02329-7

**Published:** 2025-06-16

**Authors:** Camryn Coley, Rishika Sakaria, Ranjit Philip, Shyam Sathanandam, Mark F. Weems

**Affiliations:** 1 University of Tennessee Health Science Center, Memphis, TN USA; 2 Department of Pediatrics, Division of Neonatology, Le Bonheur Children’s Hospital and the University of Tennessee Health Science Center, Memphis, TN USA; 3 Department of Pediatrics, Division of Cardiology, Le Bonheur Children’s Hospital and the University of Tennessee Health Science Center, Memphis, TN USA

**Keywords:** Outcomes research, Predictive markers, Congenital heart defects

## Abstract

**Objective:**

We aim to assess short-term cardiorespiratory outcomes of neonates after transcatheter patent ductus arteriosus (PDA) closure. We hypothesize pre-procedure respiratory severity score (RSS) ≥ 4 is associated with increased risk of post-transcatheter cardiorespiratory syndrome (PTCS).

**Study design:**

This was a retrospective study of infants who underwent transcatheter PDA closure from January 2022 to December 2022. Patients were divided by pre-procedure RSS and analyzed for the development of PTCS or hypertensive cardiorespiratory failure.

**Result:**

The study included 46 patients with a mean birthweight of 699 g and procedure weight of 1098 g. PTCS was identified in 5 (11%) patients with no differences based on pre-procedure RSS (8 vs 14%, *p* = 0.66). Post-procedure hypertension was found in 28 (61%) patients (58 vs 64%, *p* = 0.77), and occurred with respiratory failure in 12 (26%) patients (33 vs 18%, *p* = 0.32).

**Conclusion:**

Pre-procedure RSS was not associated with post-procedure outcomes. While PTCS occurred infrequently, post-procedure hypertension was common.

## Introduction

Patent ductus arteriosus (PDA) is a frequently encountered defect in infants, making up 10% of all congenital heart defects, with an incidence of two to four per 1000 live births and prevalence as high as 80% in infants weighing less than 1200 g at birth [1]. There are significant cardiac and respiratory morbidities associated with PDA in preterm infants, especially in infants born prior to 26 weeks’ gestation, and it may take several months to spontaneously close. Several non-pharmacological, pharmacological, and surgical approaches have been explored in treating a PDA, but there is neither consensus on managing extremely low birth weight (ELBW) infants with a PDA, nor consensus on which infant population will benefit from treatment [2, 3].

With improved survival rates for extremely premature infants, the prevalence of comorbidities associated with the PDA have increased, leading to the development of less-invasive treatment options such as transcatheter PDA closure (TCPC) for this population. TCPC is now considered the definitive therapy of choice in most centers in the United States, recognized as a safe and effective alternative to surgical PDA closure [3, 4].

Cardiorespiratory instability, also called post-ligation cardiac syndrome (PLCS), has been reported in 28–45% of infants after surgical PDA ligation [5–7]. In the case of TCPC, post-procedure instability has been labeled “post-transcatheter cardiorespiratory syndrome” (PTCS) [8]. While it is known that TCPC is associated with a significantly lower risk of PTCS compared to surgical ligation, limited data exists regarding the short-term cardiorespiratory changes in infants after TCPC [8–10]. The current understanding of immediate post-PDA closure instability is based on surgical ligation data. While there is not currently a reliable predictive measure for patient stability after undergoing TCPC, it has been suggested that higher levels of pre-procedure respiratory support may contribute to this phenomenon [8, 9, 11, 12]. We aim to assess the short-term effect of TCPC on the cardiorespiratory status of neonates and to identify risk factors that may be associated with respiratory worsening after PDA closure. We hypothesize that pre-procedure respiratory severity score (RSS) greater than or equal to four is associated with increased risk of PTCS.

## Materials/subjects and methods

This was a retrospective observational study performed at a single-center all-referral Level IV NICU. All consecutive infants who underwent TCPC and were on invasive mechanical ventilation prior to PDA closure from January 2022 to December 2022 were included in this study. Infants were excluded if they were not on mechanical ventilation prior to the procedure, or if there were intraprocedural complications expected to affect the level of respiratory support.

Study data were collected from the electronic medical record and managed using REDCap electronic data capture tools hosted at the University of Tennessee Health Science Center [13, 14]. Pre-procedure patient data included demographics, respiratory support, patient vital signs, PDA characteristics documented in the pre-procedure echocardiogram. RSS was calculated as the product of FiO_2_ and mean airway pressure. Pre-procedure RSS and blood pressure were calculated from the mean of all documented values from up to 24 hours before the procedure until the start of PDA closure. Post-procedure data were collected at specified intervals 1, 6, 12, 18, 24, 36, and 48 hours after completion of the PDA closure. Post-procedure RSS and blood pressure was calculated as the mean value of all documented values from the previous time point until the specified interval. Post-transcatheter cardiorespiratory syndrome (PTCS) is defined as respiratory failure (20% absolute increase in FiO_2_, 20% relative increase in mean airway pressure, or a change from conventional ventilation to high frequency) and hypotension treated with vasoactive medications within 48 hours from PDA closure [15]. Hypertension was defined as systolic blood pressure >95^th^ percentile for postmenstrual age [16]. Hypertensive respiratory failure was defined as hypertension and respiratory failure after PDA closure. Cardiorespiratory instability was defined as respiratory failure with either hypertension or hypotension. For patients discharged prior to 48 hours after TCPC, all available post-procedure data were included.

Coded data was entered into Stata 18 (StataCorp LLC., College Station, Texas) for analysis. We divided the infants into two groups based on pre-procedure RSS: RSS less than four and RSS greater than or equal to four. Student’s t-test, Wilcoxon rank-sum test and Fisher’s exact tests were used as appropriate to compare groups. Logistic regression was used to identify continuous variables associated with the development of PTCS and hypertensive cardiorespiratory failure. This study was approved by the University of Tennessee Health Science Center IRB with waiver of consent (23-09225-XP).

## Results

Fifty-one patients met inclusion criteria. Four patients were excluded because they were not on mechanical ventilation prior to the procedure. One patient was excluded due to a right atrial perforation during TCPC leading to a prolonged cardiorespiratory course. This left 46 patients for analysis. Patient demographics & pre-procedure characteristics are presented in Table 1. Except for pre-procedure RSS, there were no differences between groups when divided by RSS greater or equal to four versus less than four.

**Table 1:** Pre-procedure characteristics of infants undergoing transcatheter PDA closure.

	Total population, *n* = 46	Pre-procedure RSS < 4, *n* = 24	Pre-procedure RSS ≥ 4, *n* = 22	*p*
Gestational age, weeks, mean (sd)	25 (1.5)	25 (1.5)	25 (1.6)	0.73
Birthweight, g, mean (sd)	699 (166)	727 (169)	668 (162)	0.23
Sex				
Male*, n* (%)	21 (46)	11 (46)	10 (45)	1
Female*, n* (%)	25 (54)	13 (54)	12 (55)	
Age at procedure, days, mean (sd)	33 (11)	33 (9)	34 (13)	0.78
Weight at time of procedure, g, mean (sd)	1098 (304)	1121 (326)	1073 (283)	0.6
PMA at procedure, weeks, mean (sd)	30 (2)	30 (2)	30 (2)	0.99
RSS prior to procedure, median (IQR)	3.7 (3–5.3)	3.1 (2.7–3.3)	5.7 (4.6–7.8)	
Systemic steroids*, n* (%)	3 (6.5)	1 (4.2)	2 (9.1)	0.6
Systolic hypertension*, n* (%)	8 (17)	3 (13)	5 (23)	0.45
Received pharmacotherapy for PDA prior to procedure*, n* (%)	43 (93)	23 (96)	20 (91)	0.6
PDA characteristics				
PDA diameter, mm (sd)	2.8 (0.7)	2.8 (0.7)	2.9 (0.6)	0.7
Reversal of diastolic flow*, n* (%)	26 (57)	12 (50)	14 (64)	0.39
PDA peak velocity, m/s, mean (sd)	2.4 (0.6)	2.5 (0.6)	2.3 (0.7)	0.3
LA dilatation	42 (46)	22 (92)	20 (91)	1

*PDA* Patient ductus arteriosus, *RSS* Respiratory severity score, *PMA* Postmenstrual age, *LA* Left atrium.

Post-procedure cardiopulmonary outcomes are listed in Table 2. PTCS occurred in 11% of the total population, with no significant differences in outcomes based on pre-procedure RSS < 4 and those with RSS ≥ 4 (*p* = 0.66). Hypertensive respiratory failure was more frequent in the RSS < 4 group (33%) compared to the RSS ≥ 4 group (18%), though this difference did not reach statistical significance (*p* = 0.32). Hypotension requiring vasoactive medications and new-onset systemic hypertension were observed in 11% and 43% of infants, respectively, with no significant differences between groups. Similarly, there were no significant differences in median increases in systolic (*p* = 0.81) or diastolic (*p* = 0.86) blood pressure following PDA closure. Notably, decreased left ventricular function on the first post-procedure echocardiogram was more common in infants with RSS < 4 (33%) compared to those with RSS ≥ 4 (9%), but this did not reach statistical significance (*p* = 0.074). Outcomes were also tested with RSS ± 3, ±3.5, ±4.5 and ±5 with no differences between groups except for a 20% increase in mean airway pressure which was associated with RSS < 3 (41 vs 9%, *p* = 0.02); complete data for alternate RSS values are provided in the supplemental tables. Using logistic regression, we found that pre-procedure variables for gestational age, birthweight, procedure weight, or RSS were not associated with PTCS (Table 3). Chronological age at TCPC was nearly statistically associated with PTCS [OR 0.83 (95% CI 0.68–1.01), *p* = 0.067]. Mean age at TCPC for PTCS patients was 25 ± 7 days vs 35 ± 11 days for non-PTCS patients (*p* = 0.06). No pre-procedure variable was associated with the development of hypertensive cardiorespiratory failure or cardiorespiratory instability after TCPC.

**Table 2:** Post-procedure Cardiopulmonary characteristics of infants who underwent TCPC. 6 infants were discharged before 48 completed hours post TCPC.

	Total population, *n* = 46	Pre-procedure RSS < 4, *n* = 24	Pre-procedure RSS ≥ 4, *n* = 22	p
Post-transcatheter cardiorespiratory syndrome*, n* (%)	5 (11)	2 (8)	3 (14)	0.66
Respiratory failure*, n* (%)	15 (33)	8 (33)	7 (32)	1
Absolute increase in FiO2 ≥ 20%*, n* (%)	9 (20)	3 (13)	6 (27)	0.28
Relative increase in mean airway pressure ≥20%*, n* (%)	8 (17)	6 (25)	2 (9)	0.25
Hypotension requiring vasoactive medications*, n* (%)	5 (11)	2 (8)	3 (14)	0.66
Decreased LV function on 1^st^ post-procedure echocardiogram*, n* (%)	10 (22)	8 (33)	2 (9)	0.074
Systemic hypertension >95^th^ percentile for systolic blood pressure for age*, n* (%)	28 (61)	14 (58)	14 (64)	0.77
New-onset systemic hypertension after PDA closure*, n* (%)	20 (43)	11 (46)	9 (41)	0.77
Hypertensive respiratory failure after PDA closure*, n* (%)	12 (26)	8 (33)	4 (18)	0.32
Systemic hypertension 48 hours post-PDA closure (*n* = 40)*, n* (%)	11 (28)	5 (23)	6 (33)	0.5
Cardiorespiratory instability*, n* (%)	16 (35)	10 (42)	6 (27)	0.36
Increase in systolic blood pressure post-PDA closure, mmHg, median (IQR)	13 (5–19)	10 (6–18)	18 (2–21)	0.81
Increase in diastolic blood pressure post-PDA closure, mmHg, median (IQR)	16 (10–22)	15 (11–23)	16 (10–22)	0.86

*TCPC* Transcatheter, *PDA* closure, *RSS* Respiratory severity score, *PDA* Patent ductus arteriosus.

**Table 3:** Logistic regression models for post-transcatheter cardiorespiratory syndrome.

Variable	OR (95% CI)	p
Gestational age at birth	1.1 (0.6–2)	0.74
Birthweight	1 (0.99–1)	0.89
Weight at TCPC	1 (0.99–1)	0.3
Age at TCPC	0.83 (0.68–1.01)	0.067
PMA at TCPC	0.69 (0.38–1.3)	0.21
Pre-procedure RSS	1.1 (0.76–1.7)	0.54

*TCPC* Transcatheter PDA closure, *PDA* Patent ductus arteriosus, *PMA* Postmenstrual age, *RSS* Respiratory severity score.

Fourteen patients (30%) were on high frequency ventilation modes prior to PDA closure, 3 (13%) in the pre-procedure RSS < 4 group and 11 (50%) in the RSS ≥ 4 group (*p* = 0.006). Post-PDA closure, 13 (28%) of patients were on high frequency ventilation, 3 (13%) in the pre-procedure RSS < 4 group and 10 (45%) in the RSS ≥ 4 group (*p* = 0.01). No patient who was previously on conventional ventilation (CMV) transitioned to HFV post-TCPC, whereas one patient in the RSS ≥ 4 group was switched from HFV to CMV post-procedure. While no patient was on vasoactive medications prior to TCPC, five patients required vasoactive medications after procedure. No patients received prophylactic vasoactive medications, and all patients who required vasoactive medications after the procedure also met the definition of respiratory failure and PTCS.

**Fig. 1:**
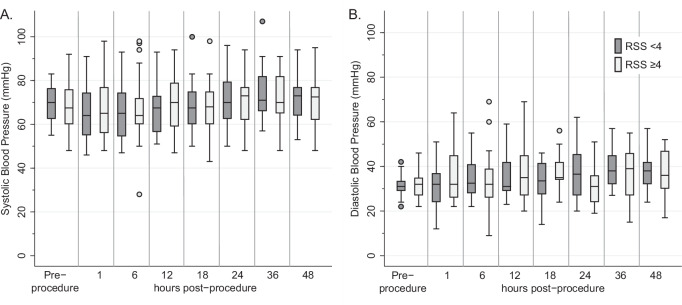
Blood pressure changes over time. Box plots are shown for (**A**) systolic blood pressure and (**B**) diastolic blood pressure prior to PDA closure and at key timepoints after closure. Infants with RSS <4 are marked in grey; infants with RSS ≥4 are marked in white. There were no significant differences between RSS groups in (**A**) systolic blood pressure or (**B**) diastolic blood pressure at any time point. *RSS* Respiratory severity score, *PDA* Patent ductus arteriosus.

Hypertension was common before PDA closure (17%) and after PDA closure (61%). Both systolic and diastolic blood pressure increased after PDA closure with no differences between groups. 71% of patients with systemic hypertension prior to PDA closure who also had post-procedure hypertension continued to have hypertension 48 hours post-procedure while only 18% of patients who developed post-procedure hypertension without first having pre-procedure hypertension still had hypertension at 48 hours post-procedure, *p* = 0.004. No patients were treated with antihypertensive medications in the 24 hours before or 48 hours after TCPC. Of the 28 patients with post-procedure hypertension, 12 (26%) also had hypertensive cardiorespiratory failure. Six patients were discharged to the referring hospital prior to 48 hours post-procedure and do not have complete blood pressure data. Pre-procedure hypertension was not associated with hypertensive cardiorespiratory failure (*p* = 0.42) or PTCS (*p* = 0.28). Figure 1 illustrates changes in systolic and diastolic blood pressure over time by pre-procedure RSS ± 4.

## Discussion

This study evaluated the short-term cardiorespiratory outcomes of neonates who underwent transcatheter PDA closure (TCPC), focusing on respiratory risk factors for post-transcatheter cardiorespiratory syndrome (PTCS). Based on RSS differences in previous studies, we chose RSS 4 as a hypothesized cutoff for increased risk of PTCS [8, 9, 11]. Although we found that pre-procedure RSS was not associated with any post-procedure outcome, we found there was a high rate of post-procedure hypertension and a low rate of PTCS. Fig. 1

Previous studies have reported that elevated pre-procedure RSS as associated with PTCS. Bischoff et al., reported in a single-center cohort of 50 infants who underwent TCPC with similar birthweight, gestational, and age at TCPC to our patient population that pre-procedure RSS was the only variable associated with post-procedure clinical instability [median 5.8 (4.1–7.9) vs 3.6 (2.9–5), *p* = 0.001] [9]. In a larger study with TCPC patients from four centers (including our center) 2017–2021, Bischoff et al. also identified higher RSS as a risk-factor for post-ligation instability [median 5.3 (4.4–8.4) vs 3.9 (2.9–5), *p* < 0.001] [8]. However, our single-center study found no association between pre-procedure RSS and the development of PTCS or hypertensive cardiorespiratory failure.

Wheeler et al. reported on a series of infants who had definitive PDA closure on conventional ventilation; they found that infants who had respiratory failure requiring a transition to high-frequency ventilation had a higher pre-procedure RSS [median 4 (3–6) vs 3.4 (2.7–4.8), *p* = 0.027) [11]. We also identified that patients with pre-procedure RSS ≥ 4 were more likely to be treated with high-frequency ventilation. However, unlike in the Wheeler study, our high RSS patients were more likely to be on high-frequency ventilation prior to TCPC, and we had no patients who were transitioned from conventional ventilation to high-frequency ventilation after TCPC. This indicates that patients with higher RSS were more likely to be managed with high-frequency ventilation, but this practice is independent of TCPC timing.

Our study found a lower overall rate of PTCS (11%) that what has been previously published. Teixeira et al. reported hemodynamic instability in 35% of infants after surgical ligation, but that group included patients treated with a fluid bolus after surgery. Excluding fluid bolus as evidence of hemodynamic instability, only 15% of the population was treated with vasoactive medications for hypotension [17]. This is slightly higher than the 11% of patients receiving vasoactive medications in our study. The previously mentioned single-center study by Bischoff et al., reported 48% PTCS after TCPC [9]. However, that used a definition of respiratory failure and/or hemodynamic support. In contrast, our definition required both respiratory failure AND hemodynamic support. When looking at individual components of the PTCS definition, the Bischoff study had 46% of infants with respiratory failure post PDA closure and 12% requiring hemodynamic support. We found 33% had respiratory failure, and 11% required hemodynamic support. This suggests there may be unrecognized practice differences that affect cardiorespiratory outcomes after TCPC. While center differences have been shown to affect PTCS and other short-term post-procedure outcomes, differences expected to affect these outcomes such as intra-procedural calcium administration or milrinone prophylaxis do not seem to affect the development of PTCS [8].

While not statistically significant, we found that patients who developed PTCS were generally younger at the time of TCPC than those who did not. A larger study population may reveal a true association between chronologic age and the development of PTCS, similar to the association reported between age and PLCS by Clyman et al. [6]. However, other studies of surgical ligation and TCPC have not found this association [6, 8, 9, 18].

An unexpected finding in our study was the high prevalence of systolic hypertension, both pre- & post-procedure. Bischoff et al. recently reported 43.6% post-procedure systolic hypertension and 49.7% among patients not treated with milrinone [8]. This is less than the 61% post-procedure systolic hypertension in our study but similar to the 43% of patients with new-onset systolic hypertension. It has been suggested that post-procedure hypertension is a result of left-ventricular diastolic dysfunction, which peaks at 24 hours after TCPC and leads to respiratory failure, and that hypertension with respiratory failure is a sign of PTCS [8]. However, this assumes that PTCS can present with dichotomous blood pressure measurements. If this is the case, then blood pressure measurement may not be a valid marker of PTCS. Our finding that most patients with hypertension did not have respiratory failure questions the relationship between post-procedure hypertension and respiratory failure. It is also notable that no clinical team felt the hypertension was significant enough to warrant treatments to lower the blood pressure. It remains unclear if post-procedure hypertension is a clinically relevant finding or a transient physiologic response to changes in cardiac loading. We suspect there are unrecognized practice standards that may affect center-specific rates of hypertension. Our practice to administer calcium chloride to all neonates during TCPC may contribute to increased rates of hypertension in our population [19, 20]. However, there may also be other factors such as ventilator management, fluid administration, and medications for sedation or analgesia that may play a role.

The prevalence of pre-procedure hypertension has not been previously reported, and we found 17% of our patients had hypertension that preceded PDA closure. Classically, the PDA has been associated with hypotension, especially diastolic hypotension [21]. Systolic blood pressure may be normal if there is adequate compensation for systemic steal, but the reason for elevated systolic blood pressure in the case of a PDA is less clear [22]. It has been reported that a PDA is a risk factor for neonatal hypertension [23, 24]. Very few of our patients were on systemic steroids pre-procedure and none were on vasoactive medications, suggesting there was no active iatrogenic contribution to hypertension. However, there are high rates of antenatal steroid administration and Indocin therapy for PDA in our referral community; these exposures have also been associated with the development of neonatal hypertension [23, 25, 26]. The clinical significance of hypertension both pre- and post-TCPC requires further investigation.

The strengths of this study include its focus on a specific population of neonates undergoing TCPC, all of whom were on invasive mechanical ventilation prior to the procedure. This ensures that we assessed a relatively homogenous group in terms of pre-procedure respiratory support, which helps to isolate the effects of the TCPC on post-procedure respiratory status.

Despite these strengths, our study has several limitations. First, this was a single-center study, retrospective study. There may be limited generalizability of the findings to other NICUs with different patient populations or procedural protocols. Second, the definition of PTCS reflects respiratory support changes and medications for hypotension; the choice of these therapies may be associated with physician practice and may not always reflect patient disease. Third, missing data from six patients discharged before 48 hours post-procedure might have underestimated the duration of hypertension, and unidentified bias may have overestimated the severity of hypertension.

Future research is needed to better understand the significance of post-procedural hypertension and to identify risk factors for PTCS. There may be center-specific practice differences or population differences that have not yet been identified. These differences may offer an opportunity for interventions that will optimize outcomes after TCPC.

## Conclusion

In this cohort of preterm infants undergoing TCPC, PTCS was infrequent and not associated with pre-procedure RSS. There was a high prevalence of systolic hypertension, both pre- and post-procedure that was generally not clinically significant.

## Supplementary information

Tables 1-7

## Data Availability

De-identified data is available from the corresponding author upon request.
