# Supplementary material for: Changes in cardiorespiratory status after transcatheter patent ductus arteriosus closure
Source: J Perinatol. 2025 Jun 16;46(3):344–8. doi: 10.1038/s41372-025-02329-7 (PMC13008771; doi:10.1038/s41372-025-02329-7)
Supplement: Supplementary file 1 — Tables 1-7 [file 41372_2025_2329_MOESM1_ESM.pdf]

Changes in Cardiorespiratory Status after Transcatheter Patent Ductus Arteriosus Closure

Camryn Coley, BA, Rishika Sakaria, MD, Ranjit Philip, MD, Shyam Sathanandam, MD, Mark F Weems, MD

SUPPLEMENTAL TABLES

sTABLE 1. Pre-procedure characteristics of infants undergoing transcatheter PDA closure grouped by RSS ±3.

	Total population, n=46	Pre-procedure RSS <3, n=12	Pre-procedure RSS ≥3, n=34	p
Gestational age, weeks, mean (sd)	25 (1.5)	25 (1.5)	25 (1.6)	0.65
Birthweight, g, mean (sd)	699 (166)	711 (144)	695 (175)	0.77
Sex				
Male, n (%)	21 (46)	6 (50)	15 (44)	0.75
Female, n (%)	25 (54)	6 (50)	19 (56)	
Age at procedure, days, mean (sd)	34 (11)	32 (7)	34 (12)	0.5
Weight at time of procedure, g, mean (sd)	1098 (304)	1037 (217)	1120 (329)	0.42
PMA at procedure, weeks, mean (sd)	30 (2)	30 (1)	30 (2)	0.4
RSS prior to procedure, median (IQR)	3.7 (3-5.3)	2.7 (2.5-2.9)	4.5 (3.6-7.1)	
Systemic steroids, n (%)	3 (6.5)	1 (8)	2 (6)	1
Systolic hypertension, n (%)	8 (17)	1 (8)	7 (21)	0.67
Received pharmacotherapy for PDA prior to procedure, n (%)	43 (93)	12 (100)	31 (91)	0.56
PDA characteristics				
PDA diameter, mm, mean (sd)	2.8 (0.7)	2.7 (0.9)	2.9 (0.6)	0.61
Reversal of diastolic flow, n (%)	26 (57)	6 (50)	20 (59)	0.74
PDA peak velocity, m/s, mean (sd)	2.4 (0.6)	2.5 (0.6)	2.3 (0.7)	0.62
LA dilatation	42 (46)	11 (92)	31 (92)	1

PDA Patient ductus arteriosus, *RSS* Respiratory severity score, *PMA* Postmenstrual age, *LA* Left atrium.

sTABLE 2. Post-procedure Cardiopulmonary characteristics of infants who underwent TCPC grouped by RSS ±3. 6 infants were discharged before 48 completed hours post TCPC.

	Total population, n=46	Pre-procedure RSS <3, n=12	Pre-procedure RSS ≥3, n=34	p
Post-transcatheter cardiorespiratory syndrome, n (%)	5 (11)	0 (0)	5 (15)	0.31
Respiratory failure, n (%)	15 (33)	5 (42)	10 (29)	0.49
Absolute increase in FiO2 ≥20%, n (%)	9 (20)	0 (0)	9 (26)	0.086
Relative increase in mean airway pressure ≥20%, n (%)	8 (17)	**5 (41)**	**3 (9)**	**0.02**
Hypotension requiring vasoactive medications, n (%)	5 (11)	0 (0)	5 (15)	0.31
Decreased LV function on 1st post-procedure echocardiogram, n (%)	10 (22)	3 (25)	7 (21)	0.71
Systemic hypertension >95th percentile for systolic blood pressure for age, n (%)	28 (61)	8 (67)	20 (59)	0.073
New-onset systemic hypertension after PDA closure, n (%)	20 (43)	7 (58)	13 (38)	0.31
Hypertensive respiratory failure after PDA closure, n (%)	12 (26)	5 (42)	7 (21)	0.25
Systemic hypertension 48 hours post-PDA closure (n=40), n (%)	11 (36)	3 (27)	8 (28)	1
Cardiorespiratory instability, n (%)	16 (35)	5 (42)	11 (32)	0.73
Increase in systolic blood pressure post-PDA closure, mmHg, median (IQR)	13 (5-19)	16 (5-20)	12 (6-19)	0.63
Increase in diastolic blood pressure post-PDA closure, mmHg, median (IQR)	16 (10-22)	20 (14-26)	15 (9-21)	0.08

TCPC Transcatheter, *PDA* closure, *RSS* Respiratory severity score, *PDA* Patent ductus arteriosus.

sTABLE 3. Pre-procedure characteristics of infants undergoing transcatheter PDA closure grouped by RSS ±3.5.

	Total population, n=46	Pre-procedure RSS <3.5, n=20	Pre-procedure RSS ≥3.5, n=26	p
Gestational age, weeks, mean (sd)	25 (1.5)	25 (1.5)	25 (1.6)	0.72
Birthweight, g, mean (sd)	699 (166)	746 (169)	663 (157)	0.09
Sex				
Male, n (%)	21 (46)	9 (45)	12 (46)	1
Female, n (%)	25 (54)	11 (55)	14 (54)	
Age at procedure, days, mean (sd)	33 (11)	32 (7)	35 (13)	0.3
Weight at time of procedure, g, mean (sd)	1098 (304)	1123 (356)	1079 (263)	0.63
PMA at procedure, weeks, mean (sd)	30 (2)	30 (2)	30 (2)	0.61
RSS prior to procedure, median (IQR)	3.7 (3-5.3)	3 (2.7-3.2)	4.9 (4.3-7.2)	
Systemic steroids, n (%)	3 (6.5)	1 (5)	2 (8)	1
Systolic hypertension, n (%)	8 (17)	2 (10)	6 (23)	0.44
Received pharmacotherapy for PDA prior to procedure, n (%)	43 (93)	20 (100)	23 (88)	0.25
PDA characteristics				
PDA diameter, mm, mean (sd)	2.8 (0.7)	2.8 (0.8)	2.9 (0.6)	0.64
Reversal of diastolic flow, n (%)	26 (57)	10 (50)	16 (62)	0.55
PDA peak velocity, m/s, mean (sd)	2.4 (0.6)	2.5 (0.6)	2.3 (0.7)	0.42
LA dilatation	42 (46)	18 (90)	24 (92)	1

PDA Patient ductus arteriosus, *RSS* Respiratory severity score, *PMA* Postmenstrual age, *LA* Left atrium.

sTABLE 4. Post-procedure Cardiopulmonary characteristics of infants who underwent TCPC grouped by RSS ±3.5. 6 infants were discharged before 48 completed hours post TCPC.

	Total population, n=46	Pre-procedure RSS <3.5, n=20	Pre-procedure RSS ≥3.5, n=26	p
Post-transcatheter cardiorespiratory syndrome, n (%)	5 (11)	2 (10)	3 (12)	1
Respiratory failure, n (%)	15 (33)	7 (35)	8 (31)	1
Absolute increase in FiO2 ≥20%, n (%)	9 (20)	2 (10)	7 (27)	0.26
Relative increase in mean airway pressure ≥20%, n (%)	8 (17)	6 (30)	2 (8)	0.062
Hypotension requiring vasoactive medications, n (%)	5 (11)	2 (10)	3 (12)	1
Decreased LV function on 1st post-procedure echocardiogram, n (%)	10 (22)	6 (30)	4 (15)	0.29
Systemic hypertension >95th percentile for systolic blood pressure for age, n (%)	28 (61)	12 (60)	16 (62)	1
New-onset systemic hypertension after PDA closure, n (%)	20 (43)	10 (50)	10 (38)	0.55
Hypertensive respiratory failure after PDA closure, n (%)	12 (26)	8 (40)	4 (15)	0.091
Systemic hypertension 48 hours post-PDA closure (n=40), n (%)	11 (28)	5 (26)	6 (29)	1
Cardiorespiratory instability, n (%)	16 (35)	10 (50)	6 (23)	0.07
Increase in systolic blood pressure post-PDA closure, mmHg, median (IQR)	13 (5-19)	10 (6-18)	15 (4-20)	0.9
Increase in diastolic blood pressure post-PDA closure, mmHg, median (IQR)	16 (10-22)	17 (13-24)	16 (9-21)	0.26

TCPC Transcatheter, *PDA* closure, *RSS* Respiratory severity score, *PDA* Patent ductus arteriosus.

sTABLE 5. Pre-procedure characteristics of infants undergoing transcatheter PDA closure grouped by RSS ±4.5.

	Total population, n=46	Pre-procedure RSS <4.5, n=29	Pre-procedure RSS ≥4.5, n=17	p
Gestational age, weeks, mean (sd)	25 (1.5)	25 (1.4)	25 (1.7)	0.26
Birthweight, g, mean (sd)	699 (166)	733 (172)	640 (139)	0.064
Sex				
Male, n (%)	21 (46)	13 (45)	8 (47)	1
Female, n (%)	25 (54)	16 (55)	9 (53)	
Age at procedure, days, mean (sd)	33 (11)	32 (10)	36 (13)	0.29
Weight at time of procedure, g, mean (sd)	1098 (304)	1117 (329)	1065 (261)	0.58
PMA at procedure, weeks, mean (sd)	30 (2)	30 (2)	30 (2)	0.97
RSS prior to procedure, median (IQR)	3.7 (3-5.3)	3.2 (2.8-3.6)	7.1 (5-7.8)	
Systemic steroids, n (%)	3 (6.5)	1 (3)	2 (12)	0.55
Systolic hypertension, n (%)	8 (17)	3 (29)	5 (29)	0.13
Received pharmacotherapy for PDA prior to procedure, n (%)	43 (93)	28 (97)	15 (88)	0.55
PDA characteristics				
PDA diameter, mm, mean (sd)	2.8 (0.7)	2.8 (0.7)	2.8 (0.6)	0.83
Reversal of diastolic flow, n (%)	26 (57)	14 (48)	12 (71)	0.22
PDA peak velocity, m/s, mean (sd)	2.4 (0.6)	2.4 (0.7)	2.3 (0.6)	0.47
LA dilatation	42 (46)	27 (93)	15 (88)	0.62

PDA Patient ductus arteriosus, *RSS* Respiratory severity score, *PMA* Postmenstrual age, *LA* Left atrium.

sTABLE 6. Post-procedure Cardiopulmonary characteristics of infants who underwent TCPC grouped by RSS ±4.5. 6 infants were discharged before 48 completed hours post TCPC.

	Total population, n=46	Pre-procedure RSS <4.5, n=29	Pre-procedure RSS ≥4.5, n=17	p
Post-transcatheter cardiorespiratory syndrome, n (%)	5 (11)	3 (10)	2 (12)	1
Respiratory failure, n (%)	15 (33)	8 (28)	7 (41)	0.52
Absolute increase in FiO2 ≥20%, n (%)	9 (20)	3 (10)	6 (35)	0.058
Relative increase in mean airway pressure ≥20%, n (%)	8 (17)	6 (21)	2 (12)	0.69
Hypotension requiring vasoactive medications, n (%)	5 (11)	3 (10)	2 (12)	1
Decreased LV function on 1st post-procedure echocardiogram, n (%)	10 (22)	8 (28)	2 (12)	0.28
Systemic hypertension >95th percentile for systolic blood pressure for age, n (%)	28 (61)	19 (65)	9 (53)	0.53
New-onset systemic hypertension after PDA closure, n (%)	20 (43)	16 (55)	4 (24)	0.064
Hypertensive respiratory failure after PDA closure, n (%)	12 (26)	10 (34)	2 (12)	0.16
Systemic hypertension 48 hours post-PDA closure (n=40), n (%)	11 (28)	6 (23)	5 (36)	0.47
Cardiorespiratory instability, n (%)	16 (35)	12 (41)	4 (24)	0.34
Increase in systolic blood pressure post-PDA closure, mmHg, median (IQR)	13 (5-19)	14 (7-18)	11 (1-20)	0.28
Increase in diastolic blood pressure post-PDA closure, mmHg, median (IQR)	16 (10-22)	16 (11-23)	16 (10-21)	0.49

TCPC Transcatheter, *PDA* closure, *RSS* Respiratory severity score, *PDA* Patent ductus arteriosus.

sTABLE 6. Pre-procedure characteristics of infants undergoing transcatheter PDA closure grouped by RSS ±5.

	Total population, n=46	Pre-procedure RSS <5, n=33	Pre-procedure RSS ≥5, n=13	p
Gestational age, weeks, mean (sd)	25 (1.5)	25 (1.5)	25 (1.7)	0.47
Birthweight, g, mean (sd)	699 (166)	719 (169)	648 (152)	0.19
Sex				
Male, n (%)	21 (46)	15 (45)	6 (46)	1
Female, n (%)	25 (54)	18 (55)	7 (54)	
Age at procedure, days, mean (sd)	33 (11)	32 (10)	38 (13)	0.13
Weight at time of procedure, g, mean (sd)	1098 (304)	1101 (321)	1091 (268)	0.92
PMA at procedure, weeks, mean (sd)	30 (2)	30 (2)	30 (2)	0.55
RSS prior to procedure, median (IQR)	3.7 (3-5.3)	3.3 (2.8-4.1)	7.2 (6.7-8.6)	
Systemic steroids, n (%)	3 (6.5)	2 (6)	1 (8)	1
Systolic hypertension, n (%)	8 (17)	6 (18)	2 (15)	1
Received pharmacotherapy for PDA prior to procedure, n (%)	43 (93)	31 (94)	12 (92)	1
PDA characteristics				
PDA diameter, mm, mean (sd)	2.8 (0.7)	2.8 (0.7)	2.9 (0.6)	0.87
Reversal of diastolic flow, n (%)	26 (57)	17 (52)	9 (69)	0.34
PDA peak velocity, m/s, mean (sd)	2.4 (0.6)	2.4 (0.6)	2.3 (0.7)	0.67
LA dilatation	42 (46)	31 (94)	11 (85)	0.57

PDA Patient ductus arteriosus, *RSS* Respiratory severity score, *PMA* Postmenstrual age, *LA* Left atrium.

sTABLE 7. Post-procedure Cardiopulmonary characteristics of infants who underwent TCPC grouped by RSS ±5. 6 infants were discharged before 48 completed hours post TCPC.

	Total population, n=46	Pre-procedure RSS <5, n=33	Pre-procedure RSS ≥5, n=13	p
Post-transcatheter cardiorespiratory syndrome, n (%)	5 (11)	4 (12)	1 (8)	1
Respiratory failure, n (%)	15 (33)	10 (30)	5 (38)	0.73
Absolute increase in FiO2 ≥20%, n (%)	9 (20)	5 (15)	4 (31)	0.25
Relative increase in mean airway pressure ≥20%, n (%)	8 (17)	6 (18)	2 (15)	1
Hypotension requiring vasoactive medications, n (%)	5 (11)	4 (12)	1 (8)	1
Decreased LV function on 1st post-procedure echocardiogram, n (%)	10 (22)	8 (24)	2 (15)	0.7
Systemic hypertension >95th percentile for systolic blood pressure for age, n (%)	28 (61)	22 (67)	6 (46)	0.32
New-onset systemic hypertension after PDA closure, n (%)	20 (43)	16 (48)	4 (31)	0.34
Hypertensive respiratory failure after PDA closure, n (%)	12 (26)	11 (33)	1 (8)	0.14
Systemic hypertension 48 hours post-PDA closure (n=40), n (%)	11 (28)	7 (24)	4 (36)	0.46
Cardiorespiratory instability, n (%)	16 (35)	14 (42)	2 (15)	0.1
Increase in systolic blood pressure post-PDA closure, mmHg, median (IQR)	13 (5-19)	14 (7-18)	11 (1-20)	0.52
Increase in diastolic blood pressure post-PDA closure, mmHg, median (IQR)	16 (10-22)	16 (11-23)	16 (10-21)	0.38

TCPC Transcatheter, *PDA* closure, *RSS* Respiratory severity score, *PDA* Patent ductus arteriosus.
